# Predicting Discharge Location among Low-Energy Hip Fracture Patients Using the Score for Trauma Triage in the Geriatric and Middle-Aged (STTGMA)

**DOI:** 10.1155/2018/9793435

**Published:** 2018-11-18

**Authors:** Sanjit R. Konda, Hesham Saleh, Ariana Lott, Kenneth A. Egol

**Affiliations:** Department of Orthopedic Surgery, NYU Langone Medical Center, 301 East 17th Street, New York, NY 10003, USA

## Abstract

Patterns of discharge location may be evident based on the “sickness” profile of the patient. This study sought to evaluate the ability of the STTGMA tool, a validated mortality risk index for middle-aged and geriatric trauma patients, to predict discharge location in a cohort of low-energy elderly hip fracture patients, with successful discharge planning measured by readmission rates. Low-energy hip fracture patients aged 55 years and older were prospectively followed throughout their hospitalization. On initial evaluation in the Emergency Department, each patient's age, comorbidities, injury severity, and functional status were utilized to calculate a STTGMA score. Discharge location was recorded with the primary outcome measure of an unsuccessful discharge being readmission within 30 days. Patients were risk stratified into minimal-, low-, moderate-, and high-risk STTGMA cohorts. A p-value of <0.05 was considered significant for all statistical tests. 408 low-energy hip fractures were enrolled in the study with a mean age of 81.3±10.6 years. There were 214 (52.5%) intertrochanteric fractures, 167 (40.9%) femoral neck fractures, and 27 (6.6%) subtrochanteric femur fractures. There was no difference in readmission rates within STTGMA risk cohorts with respect to discharge location; however, among individual discharge locations there was significant variation in readmission rates when patients were risk stratified. Overall, STTGMA risk cohorts appeared to adequately risk-stratify readmission with 3.5% of minimal-risk patients experiencing readmission compared to 24.5% of moderate-risk patients. Specific cohorts deemed high-risk for readmission were adequately identified. The STTGMA tool allows for prediction of unfavorable discharge location in hip fracture patients. Based on observations made via the STTGMA tool, improvements in discharge planning can be undertaken to increase home discharge and to more closely track “high-risk” discharges to help prevent readmissions.

## 1. Introduction

According to national projections by the US Census Bureau, the older population of the United States will undergo considerable growth in the upcoming years [1]. Current estimations report that the geriatric population, aged 65 years and older, will increase from 43.1 million in 2012 to 83.7 million in 2050 [1]. It is estimated that they will account for over 20% of the general population [2]. Trauma is currently the fifth leading cause of death in older adults. In 2050, the older population will account for approximately 40% of all trauma cases [3].

Hip fractures are common in the middle-aged and geriatric trauma population. Currently, more than 250,000 hip fractures occur each year in the United States, with that estimate expected to increase to 840,000 by 2040 [4]. Hip fractures are associated with significant morbidity, mortality, and costs [5]. In fact, hip fractures account for 14% of all fractures yet comprise 72% of overall fracture care costs [6]. Given the expected increase in the older population and serious health and financial consequences associated with hip fractures in this population, proper management of these patients is imperative.

Interdisciplinary care, consisting of geriatric consultations, discharge planning, and rehabilitation has been shown to improve functional capacity and reduce mortality after hip fracture surgery [7–9]. In conjunction with a multidisciplinary approach, having defined postacute care pathways reduces costs and utilization of hospital resources [10]. Although patient care involves several important aspects, this study focuses on discharge planning.

Early discharge planning reduces lengths of stay, costs, and utilization of hospital resources, while decreasing patient mortality and improving functional outcomes [11]. Thus, orthopaedic surgeons should identify patients who are candidates for discharge home versus those who will require rehabilitation services or skilled levels of care early in the admission to facilitate discharge planning [12]. Certain factors, such as advanced age, preoperative motor ability, preexisting comorbidities, and cognition have been shown to affect discharge location [12, 13]. However, there are few methodologies that assist orthopaedic surgeons to systematically predict the discharge locations of their patients.

The Score for Trauma Triage in the Geriatric and Middle-Aged (STTGMA) is a novel inpatient mortality risk tool developed and validated in the National Trauma Databank as well as at our institution as a reliable tool for prediction of inpatient mortality [14, 15]. The purpose of this study is to investigate whether STTGMA scores, calculated upon initial admission in the Emergency Department, can be used to predict patients who will require postacute facility care when discharged and can therefore provide a valuable tool to guide discharge planning for patients with low-energy hip fractures with successful discharge planning measured by readmission rates.

## 2. Materials and Methods

From 10/1/2014 to 9/30/2016, all patients aged 55 years and older who were admitted with a hip fracture AO/OTA fracture classification of 31-A, 31-B, and (32-A(1-3).1, 32-B(1-3).1, 32-C(1-3).1) were enrolled in this IRB-approved study and followed prospectively at 2 level 1 trauma centers and 1 academic tertiary-care center. “Middle-aged” was defined as patients aged 55-64 years old, while “geriatric” as ages ≥ 65 years. Only low-energy mechanisms of injury, defined as ground-level falls up to two levels of stair height, were included [14]. Exclusion criteria were any patients that died within the Emergency Department setting prior to admission.

Upon initial evaluation in the Emergency Department, basic demographics and all STTGMA variables were collected to provide information regarding each patient's injury, health, and functional status. Glasgow Coma Scale (GCS) and Abbreviated Injury Severity (AIS) for the head/neck and chest were utilized for injury status. Charlson Comorbidity Index (CCI) represented the patient's health status. Their ambulatory capability (community, household, nonambulatory) represented their functional status. Note that for ambulatory capacity, patients were considered a community ambulator if they spent ≥ 50% of their time ambulating outside their primary residence, a household ambulatory if they spent ≥ 50% of their time ambulating within their residence, and a nonambulator if they were unable to ambulate without assistance. These variables, summarized in Table 1, were utilized to calculate a STTGMA score, which provides the predicted risk (0-100%) of inpatient mortality during the index hospitalization. All STTGMA variables were collected by junior year orthopaedic surgery residents who had been trained in STTGMA score calculation via a standardized online 20 minute tutorial [16].

Patients were prospectively followed throughout their admission. Discharge location was determined by the admitting physician in consultation with a social worker and case manager, all of whom were blinded to patient's STTGMA score. Discharge locations were collected and included discharge to home, acute inpatient rehabilitation facility, skilled nursing facility (SNF) as well as discharges to hospice, long-term care facilities (LTCF), transfer to another acute hospital, and death during index hospitalization. Readmission data within 30 days from discharge was obtained. To make this tool clinically useful for guiding early discharge planning, patients were divided into minimal-risk (<0.4%), low-risk (0.4-1.5%), moderate-risk (1.5-5.0%), and high-risk (>5% STTGMA score) patients. These risk groups were compared considering a p-value < 0.05 as significant for all statistical tests.

## 3. Results

A total of 408 consecutive patients with low-energy hip fractures were included in the cohort and prospectively followed throughout their index hospitalization. No patients were lost to follow-up. The mean age at the time of injury was 81.3 ± 10.6 years. Of this cohort, 167 (40.9%) sustained femoral neck fractures (31-B), 214 (52.5%) intertrochanteric fractures (31-A), and 27 (6.6%) subtrochanteric fractures (32-A(1-3).1, 32-B(1-3).1, 32-C(1-3).1). 18 patients (4.4%) patients were managed nonoperatively, and 9 patients (2.2%) died during their index admission. 29 (7.1%) underwent total hip arthroplasty, 77 (18.9%) underwent hemiarthroplasty, and 284 (69.9%) underwent open reduction internal fixation (ORIF) (Table 2).

With regard to injury status, the mean GCS was 14.9 ± 0.8, the mean AIS Head/Neck was 0.04 ± 0.24, and the mean AIS Chest was 0.03 ± 0.19, With regard to health status the mean CCI was 1.4 ± 1.6. With regard to functional status, the mean age was 80.3 ± 10.6 and the majority of patients were either community (290, 71.1%) or household (91, 22.3%) ambulators.  Table 3 summarizes these results in more detail. Utilizing these variables, the mean STTGMA score for the entire cohort was 1.7%  ± 5.1%. 226 patients (55.4%) were included in the minimal-risk cohort (STTGMA score <0.4%), 102 patients (25.0%) were included in the low-risk cohort (STTGMA score 0.4%-1.5%), 53 patients (13.0%) were included in the moderate-risk cohort (STTGMA score 1.5%-5.0%), and 27 patients (6.6%) were included in the high-risk cohort (STTGMA score >5.0%). There was no difference in mean STTGMA score between the fracture pattern cohorts; however, as expected, there was a significant difference in mean STTGMA score between the procedure-specific cohorts with patients in the total hip arthroplasty group having a lower mean STTGMA score than those in the hemiarthroplasty or ORIF group (0.3±0.6% versus 2.1±5.9% versus 1.1±2.3%; p=0.024).

Of the 408 patients, 84 (20.6%) were discharged to home, 61 (15.0%) to an acute rehabilitation facility and 245 (60.0%) to a skilled nursing facility. There were 9 (2.2%) patients who died during the index hospitalization, 2 (0.5%) patients who was transferred to an outside hospital, 2 (0.5%) patients who were transferred to LTCF, and 5 (1.2%) patients who were transferred to hospice care. There were statistically significant differences in discharge patterns between the risk groups (p<0.005). While 63 patients in the minimal-risk cohort (27.9%) were discharged home, this percentage decreased significantly in the higher risk groups. The percentage of patients discharged to skilled nursing facilities also increased in the higher risk groups with 77.4% of the moderate-risk cohort discharged to SNF compared to 52.2% of the minimal-risk cohort (Table 4).

Overall, STTGMA risk cohorts also appeared to adequately risk-stratify readmission with 3.5% of minimal-risk patients requiring readmission compared to 24.5% of moderate-risk patients. Specific cohorts were deemed high-liability for readmission including low and moderate-risk STTGMA patients discharged home (28.5% and 33.3% readmission rate, respectively); moderate- and high-risk STTGMA patients discharged to SNF (21.9% and 14.3% readmission rate, respectively) and low-, moderate-, and high-risk patients discharged to AR (18.2%, 33.3%, and 50% readmission rates, respectively). However, within each cohort, discharge location (home, SNF, acute rehab) did not have an impact on readmission risk for each risk group (Table 5).

## 4. Discussion

The Score for Trauma Triage in the Geriatric and Middle-Aged is able to risk-stratify low-energy hip fracture patients with respect to discharge location and risk of readmission within 30 days after discharge. STTGMA can also be used to more closely track “high-risk” discharges to prevent readmissions. This analysis also demonstrates that improvements in discharge planning can be undertaken to encourage more home discharge in hip fracture patients as there were no differences in readmission risk based on discharge location within each risk group.

An important aspect of the STTGMA tool is that it is calculated from variables collected in the Emergency Department upon initial admission. Thus, STTGMA may be utilized as a clinical risk tool by orthopaedic surgeons to better guide patient care early within the hospitalization in preparation of discharge. The results of this study suggest that patients in the higher risk groups have a higher incidence of discharge to SNFs, while patients in the minimal-risk cohort are more likely to be discharged to home or acute inpatient rehabilitation. Based on these results we recommend that orthopaedic surgeons, along with their multidisciplinary team, commence early discharge planning on either hospital day 0 or 1 using the STTGMA tool to guide planning to home, acute inpatient rehabilitation or to SNF based on their STTGMA result. Early discharge planning may decrease patient mortality and improve their functional outcomes [14]. In addition, it will lead to reductions in inpatient lengths of stay, hospital costs, and utilization of hospital resources [14]. Furthermore, hospitals may use the STTGMA tool to more closely track the “high-risk” discharges as we identified in this study to prevent readmission including low- and moderate-risk STTGMA patients discharged home, moderate- and high-risk STTGMA patients discharged to SNF, and low-, moderate-, and high-risk patients discharged to acute rehab.

Our results demonstrated that STTGMA was able to associate patients, based on historical discharge location patterns, into a home, acute inpatient rehabilitation, and SNF category. This ability is important given the current shift in hip fracture reimbursement to a bundled payment model with the goal of improvement in the quality of care of these patients. The average Medicare payment for the initial rehabilitation stay for hip fracture patients is approximately $15,183 [17]. It is estimated that rehabilitation provides an additional 622 days of life. Estimations report that the average Medicare payment for such a patient is approximately $78 per day for two years following hospitalization [13]. Taking this cost information into account, increased savings may be realized if patients can be safely discharged home instead of discharged to acute inpatient rehabilitation or a subacute nursing facility. The use of STTGMA allows for identification of those patients who historically would have been discharged to acute inpatient rehabilitation or a subacute nursing facility but who may be safely discharged home with home-based physical therapy. This is an area open for future study.

There are several limitations to our study. First, although this study included several different types of hospitals, all hospitals are in an urban environment and may not be representative of other institutions throughout the country. Furthermore, our discharge rates to home and to acute inpatient rehabilitation are greater than those typically cited in the hip fracture population [18]. One possible reason for the high rate of home discharge during the study period is that all hospitals included in this study had a policy to encourage home discharge for all patients with acceptable family support. There was no standardized protocol to encourage home discharge, however, and this decision was made via shared decision-making process involving the patient/family and attending physician. With respect to the high rate of acute rehabilitation discharges, one possible explanation is that at two of the hospitals included in the study, there is an acute rehab facility within the hospital making acute rehab discharge often a simpler process than a discharge to a skilled nursing facility or home. Second, information regarding patients' race, socioeconomic status, and education level were not collected; investigating these variables may reveal confounding factors. In future studies, we plan to collect this data to determine its effect on the correlation found between STTGMA score and discharge location.

## 5. Conclusions

This novel scoring system, the Score for Trauma Triage in the Geriatric and Middle Aged, has the capacity to identify hip fracture patients who are likely to be discharged to a postacute care facility after inpatient hospitalization. Thus, it is a valuable clinical risk tool for orthopaedic surgeons in guiding patient care and early preparation of discharge planning for at-risk patients. Early discharge planning has multiple proven benefits for both the patient and the healthcare system.

## Figures and Tables

**Table 1:** Summary of variables utilized to calculate a low-energy STTGMA score.

**Injury Status**	**Health Status**	**Functional Status**
Low Energy	CCI	Ambulatory capacity
GCS		
AIS Head/Neck		
AIS Chest		

**Table 2:** Demographics, summary of the injuries sustained (anatomic location and AO/OTA fracture classification), and procedure performed on the cohort of 408 hip fracture patients.

	Total Cohort (n=408 patients)

Age (years) (mean ± SD)	81.3 ± 10.6

Gender (female) (n,%)	286 (70.1%)

**Fracture Type:**	

Intertrochanteric Fractures (31-A)	214 (52.5%)

Femoral Neck Fractures (31-B)	167 (40.9%)

Subtrochanteric Fractures (32-A(1-3).1, 32-B(1-3).1, 32-C(1-3).1)	27 (6.6%)

**Procedure:**	

Open Reduction Internal Fixation	284 (69.6%)

Hemiarthroplasty	77 (18.9%)

Total Hip Arthroplasty	29 (7.1%)

Non-operative	18 (4.4%)

**Table 3:** Summary of the injury, health, and functional status variables utilized to calculate STTGMA scores.

**Injury Status**			

*Glasgow Coma Scale*		*AIS Head/Neck*	

GCS 15	380 (93.1%)	AIS 0	394 (96.6%)
GCS 14	17 (4.2%)	AIS 1	12 (2.9%)
GCS 13	5 (1.2%)	AIS 2	1 (0.2%)
GCS 12	2 (0.5%)	AIS 3	1 (0.2%)
GCS 11	2 (0.5%)	Mean ±SD	0.04 ± 0.24
GCS 6	1 (0.2%)		
GCS 5	1 (0.2%)		
Mean ± SD	14.85 ± 0.80		

*AIS Chest*			

AIS 0	396 (97.1%)		
AIS 1	11 (2.7%)		
AIS 2	1 (0.2%)		
Mean ± SD	0.03 ± 0.19		

**Health Status**			

*Charlson Comorbidity Index*		*Age *	

0	146 (35.8%)	55-59	17 (4.2%)
1	108 (26.5%)	60-69	59 (14.5%)
2	80 (19.6%)	70-79	87 (21.3%)
3	36 (8.8%)	80-89	151 (37.0%)
4	15 (3.7%)	90-99	93 (22.8%)
5	4 (1.0%)	>100	1 (0.2%)
6	13 (3.2%)	Mean ± SD	81.3 ± 10.6 years
7	4 (1.0%)		
8	2 (0.5%)		
Mean ± SD	1.42 ± 1.62		

**Functional Status**			

*Ambulatory Status*			

Community	290 (71.1%)		
Household	91 (22.3%)		
Non-ambulator	27 (6.6%)		

**Table 4:** Discharge location patterns for minimal-, low-, moderate-, and high-risk cohorts. Percentages reflect proportion of each respective risk cohort discharged to each location.

*Location*	*Minimal Risk Cohort (n=226)*	*Low Risk Cohort (n=102)*	*Moderate Risk Cohort (n=53)*	*High Risk Cohort (n=27)*	*Total Cohort (n=408)*
Home	63 (27.9%)	12 (11.8%)	5 (9.4%)	4 (14.8%)	84 (20.6%)

Acute Rehab	41 (18.1%)	15 (14.7%)	3 (5.7%)	2 (7.4%)	61 (15.0%)

Skilled Nursing Facility	118 (52.2%)	72 (70.6%)	41 (77.4%)	14 (51.9%)	245 (60.0%)

Hospice	1 (0.4%)	1 (1.0%)	0 (0.0%)	3 (11.1%)	5 (1.2%)

LTCH	1 (0.4%)	0 (0.0%)	1 (1.9%)	0 (0.0%)	2 (0.5%)

Death	2 (0.9%)	1 (1.0%)	2 (3.8%)	4 (14.8%)	9 (2.2%)

Transfer to Acute Hospital	0 (0.0%)	1 (1.0%)	1 (1.9%)	0 (0.0%)	2 (0.5%)

**Table 5:** Length of stay and readmission rate for minimal-, low-, moderate-, and high-risk cohorts stratified by discharge location. Percentages reflect proportion of each respective risk cohort readmitted. P-value^a^ analyzes differences in readmission rates by discharge location (within a specific risk group); P-value^b^ analyzes differences in readmission rates, length of stay, and discharge location among risk groups.

	*Minimal Risk Cohort (n=226)*	*Low risk Cohort (n=102)*	*Moderate Risk Cohort (n=53)*	*High Risk Cohort (n=27)*	*Total *	*p-value*_*b*_
Length of Stay (days) (mean±SD)	7.4 ± 4.5	8.4 ± 6.6	9.0 ± 5.0	8.5±5.2	8.0±5.2	0.128

Readmission rate N (%)	8 (3.5%)	7 (6.9%)	13 (24.5%)	3 (11.1%)	31 (7.6%)	<0.005
Home N (%)	4 (6.3%)	2 (16.7%)	2 (40.0%)	0 (0.0%)	8 (9.5%)	<0.005
Acute Rehab N (%)	1 (2.4%)	2 (13.3%)	1 (33.3%)	1 (50.0%)	5 (8.2%)	<0.005
Skilled Nursing Facility N (%)	3 (2.5%)	3 (4.2%)	10 (24.4%)	2 (14.3%)	18 (7.3%)	<0.005
p-value^a^	0.385	0.174	0.729	0.268	0.814	

## Data Availability

The data used to support the findings of this study are included within the article.
